# Pitfalls in the quantitative imaging of glutathione in living cells

**DOI:** 10.1038/s41467-018-04035-9

**Published:** 2018-04-23

**Authors:** Cristina Cossetti, Gianna Di Giovamberardino, Rossella Rota, Anna Pastore

**Affiliations:** 1 Laboratory of Epigenetics of Pediatric Sarcomas, Division of Hematology/Oncology, Children’s Hospital and Research Institute “Bambino Gesù”, Viale di San Paolo 15, Rome, 00146 Italy; 2 Laboratory of Molecular Genetics and Functional Genomics, Division of Genetic and Rare Disease, Children’s Hospital and Research Institute “Bambino Gesù”, Viale di San Paolo 15, Rome, 00146 Italy

Biothiols have crucial roles in maintaining redox homeostasis in biological systems. Glutathione (GSH) and its precursor, cysteine (Cys), are the two most abundant low-molecular weight thiols in living cells, and their intracellular abnormal levels are associated with diseases^1^. GSH is a ubiquitous thiol-containing tripeptide, which has a central role in cell biology. It is implicated in the cellular defense against xenobiotics and naturally occurring deleterious compounds, such as free radicals and hydroperoxides^2^. The ratio of GSH to its oxidized form (glutathione disulfide, GSSG) is an indicator of the redox state of the cell, and the alteration of this ratio can lead to a number of human pathologies^3^. Consequently, the assessment of abnormal levels of thiol-containing substances in biological systems may provide valuable information for the early diagnosis of some diseases^2,3^.

Recently, Jiang et al.^4^ reported the characterization of a fluorescent probe for quantitative real-time imaging of GSH in living cells. This probe is commercially available as RealThiol (RT, to be used for calibrations) and RealThiol AM Ester (RT-AM, to be used with cells) GSH Detection Probe (Kerafast, Boston, MA, USA). Authors performed an impressive number of experiments, aimed to demonstrate that RT preferentially reacts with GSH under physiological conditions. In contrast to that reported by Jiang et al.^4^, we show here that RT is able to react with both GSH and Cys (Fig. 1a, b).

**Fig. 1:**
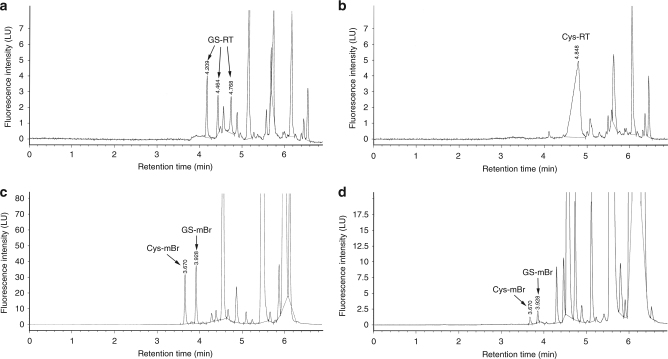
RT probe reacts with both Cys and GSH in physiological conditions. **a** Reaction of 200 μM of GSH with RT probe. In our experience, such concentrations of GSH are often found in various biological specimen, such as tissues or cells. GS-RT separation resulted in three almost symmetrical peaks at 4.209, 4.464, and 4.768 min of retention time, respectively. **b** Reaction of 200 μM of Cys with RT probe. This Cys concentration is often found in various biological samples. The separation of Cys-RT resulted in one well-resolved symmetrical peak at 4.848 min retention time, with a response in terms of fluorescence intensity higher than those of GS-RT. **c** A representative chromatogram of thiols determination in mouse hippocampus cells. In these conditions, the levels of Cys (3.670 min retention time) and GSH (3.928 min retention time) are almost identical (110 and 125 μM, respectively). **d** Chromatogram of thiols determination in H_2_O_2_-treated HeLa cells. Levels of Cys and GSH are similar (20 and 28 μM, respectively)

It should be noted that analytical methods for thiols assessment typically involve separation steps, and one of the most used method for the separation of biothiols is high performance liquid chromatography (HPLC) coupled either with fluorescence^5^ (HPLC-FD), mass-spectrometry^6^ (HPLC-MS), or tandem mass-spectrometry detection^7^ (HPLC-MSMS). Although Jiang et al.^4^ used HPLC-MS for some of the GSH measurements, they do not show whether Cys is present or not in their HeLa cells before and after treatments with H_2_O_2_. In addition, they quantify thiols by HPLC-MS after reaction with *N*-methylmaleimide (NMM), using 100 μM NMM in some experiments, and 1 mM NMM in others. NMM is identical to the other chemical used for –SH groups alkylation, *N*-ethylmaleimide (NEM). Although NEM reacts with thiols by a very fast Michael addition reaction at neutral or slightly acidic pH, this reaction is reversible, and an excess of NEM should be used to avoid the possible migration of the maleimide group among different thiols^3^. This is not accomplished by Jiang et al.^4^, that use 100 μM or 1 mM NMM. These concentrations are insufficient to be sure that all thiols present are blocked, especially in cells where GSH levels are higher than 1 mM[3].

Over the years, great efforts have been devoted to develop reaction-based fluorescent sensors for the detection of thiols in living systems^8^. Nonetheless, Yin et al.^8^ just highlight that despite a large number of thiol-reactive reagents and probes have been commercially available for many years, filling an entire chapter of the Molecular Probes Handbook^9^, most of them are non-selective, forming covalent adducts with any sulfhydryl-containing molecules. Notably, it is important to realize that such universal thiol-reactive probes are sometimes marketed or reported in the literature as GSH (or Cys) selective. This erroneous interpretation is often based on the assumption that GSH is believed to be the most abundant thiol in cells, and any probe signaling from an interaction with Cys or other thiols would be negligible. This may be true, but it has been clearly demonstrated that their concentrations varies not only depending on the cell type and treatments, but also during cell proliferation in comparison with quiescent or not dividing cells^10^. Indeed, in our more that ten-year experience on thiols measurements in various cells and tissues^11–15^, the concentration of GSH has been often well below 1 mM, and comparable to that of Cys (Fig. 1c). This is also true in H_2_O_2_-treated HeLa cells, where GSH and Cys levels become almost identical after treatment (Fig. 1d).

On the basis of the literature reports and on our data, we believe that an unaware probe end-user could encounter artificially attributed high values of GSH that are arising instead from both GSH and Cys, including Cys-protein residues. Because of the possible presence of high Cys levels, the use of RT probe for GSH quantification must be applied cautiously. The reactivity of RT probe towards GSH is obviously not questioned, and the use of this probe could be useful after verifying, possibly by HPLC, that in an experimental system the amount of GSH is at least one order of magnitude greater than that of Cys, both before and after any treatment aimed to change thiols levels.

## Methods

### Cell culture and treatment

HeLa cells were kindly provided by Lucio Miele, MD (Louisiana State University, Stanley S. Scott Cancer Center, New Orleans, LA, USA), and grown in RPMI medium, 10% FBS, supplemented with 1% penicillin/streptomycin and 1% l-glutamine (All from Invitrogen, Carlsbad, CA, USA) in a humidified atmosphere of 5% CO_2_/95% air. All the used aliquots were routinely tested for mycoplasma. HeLa cells were treated for 24 h with 100 µM H_2_O_2_ (Sigma, St Louis, MO, USA) in the culture medium and cell pellets were then collected and analyzed as reported below.

### HPLC determination of Cys and GSH with RT probe derivatization

Reaction of standard solution of 200 μM of Cys or GSH with RT probe was carried out following the manufacturer protocol (Kerafast, Boston, MA, USA). Overall, 10 μL of the reaction mixture was injected into a 150 × 4.6 mm Hypersil™ ODS column (Thermo Fisher Scientific, Waltham, MA USA) equilibrated with 30 mmol/L ammonium nitrate and 40 mmol/L ammonium formate buffer, pH 3.6 (buffer A). The thiols were eluted from the column with a 6-min gradient of acetonitrile (buffer B) (0–4 min, 0–30% buffer B; 4–5 min, 30–100% buffer B; 5–6 min, 100% buffer B) at a flow rate of 1.5 mL/min at ambient temperature. The HPLC was an Agilent 1290 (Agilent Technologies, Santa Clara, CA, USA), equipped with a fluorescence detector operating at excitation wavelengths of 405 and 488 nm, and emission wavelength of 499 nm.

### HPLC determination of Cys and GSH with monobromobimane (mBr) derivatization

The cells were sonicated three times for 2 s in 100 µL of 0.1 M potassium phosphate buffer (pH 7.2). After sonication, 50 μL of 12% sulfosalicylic acid were added, and the Cys and GSH content in the acid-soluble fraction was determined (free Cys and free GSH). To measure cystine and oxidized glutathione (GSSG), cells were sonicated three times for 2 s in 0.1 mL of 0.1 M potassium phosphate buffer (pH 7.2) containing 10 mM *N*-ethylmaleimide. Cys and GSH levels were calculated by subtracting cystine and GSSG concentrations from free Cys and free GSH values. The derivatization procedure was performed as follows: 30 µL of 4 mol/L NaBH_4_, 20 µL of 2 mmol/L EDTA/DTT, 10 µL of 1-octanol and 20 µL of 1.8 mol/L HCl were placed in the derivatization vial containing 30 µL of sample. After incubation for 3 min, 100 µL of 1.5 mol/L *N*-ethylmorpholine buffer, pH 8.0, 400 μL of distilled water, and 20 µL of 25 mmol/L bromobimane were added. After additional 3-min incubation, 40 μL of acetic acid was added, and 20 μL of the reaction mixture was injected into a 150 × 4.6 mm Hypersil™ ODS column (Thermo Fisher Scientific, Waltham, MA, USA) equilibrated with 30 mmol/L ammonium nitrate and 40 mmol/L ammonium formate buffer, pH 3.6 (buffer A). The thiols were eluted from the column with a 6-min gradient of acetonitrile (buffer B) (0–4 min, 0–30% buffer B; 4–5 min, 30–100% buffer B; 5–6 min, 100% buffer B) at a flow rate of 1.5 mL/min at ambient temperature. The HPLC was an Agilent 1290 (Agilent Technologies, Santa Clara, CA, USA), equipped with a fluorescence detector operating at excitation wavelengths of 390 nm, and emission wavelength of 478 nm.

### Data availability

The data that support the findings of this study are available from the corresponding author upon reasonable request.
